# Supplementary material for: The interactome of intact mitochondria by cross-linking mass spectrometry provides evidence for coexisting respiratory supercomplexes
Source: Mol Cell Proteomics. 2017 Dec 8;17(2):216–32. doi: 10.1074/mcp.RA117.000470 (PMC5795388; doi:10.1074/mcp.RA117.000470)
Supplement: Supplemental Data [file supp_RA117.000470_133922_0_supp_23978_fzffwf.zip › spectra_annotation/mito_spectra_annotation/319-1-7-1-7-1.pdf]

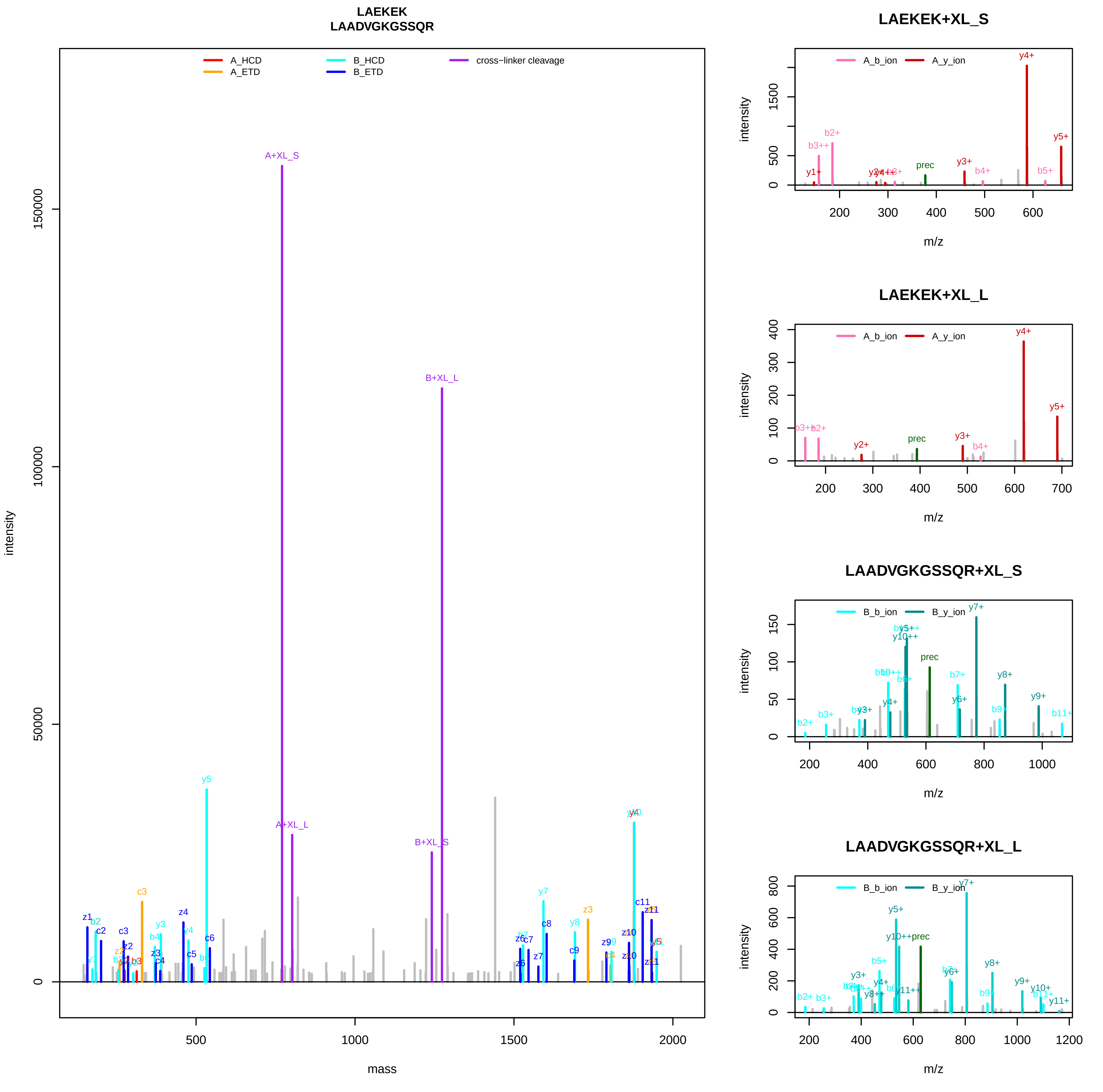

LAEKEK
LAADVGKGSSQR
A_HCD
A_ETD
B_HCD
B_ETD
cross−linker cleavage
A+XL_S
B+XL_L
A+XL_L
B+XL_S
y5
intensity
mass
0
50000
100000
150000
500
1000
1500
2000
LAEKEK+XL_S
A_b_ion
A_y_ion
y4+
y5+
b2+
b3++
y1+
prec
y3+
b4+
b5+
m/z
LAEKEK+XL_L
LAADVGKGSSQR+XL_S
B_b_ion
B_y_ion
y7+
y10++
y8+
y9+
b11+
LAADVGKGSSQR+XL_L
